# The effect of γ-FeOOH on enhancing arsenic adsorption from groundwater with DMAPAAQ + FeOOH gel composite

**DOI:** 10.1038/s41598-019-48233-x

**Published:** 2019-08-15

**Authors:** Syed Ragib Safi, Kiyotaka Senmoto, Takehiko Gotoh, Takashi Iizawa, Satoshi Nakai

**Affiliations:** Department of Chemical Engineering, Hiroshima University, 1-4-1 Kagamiyama, Higashi Hiroshima, Hiroshima, 739-8527 Japan

**Keywords:** Gels and hydrogels, Pollution remediation, Chemical engineering

## Abstract

Arsenic contamination of groundwater is a serious concern worldwide. The research gaps in removing arsenic are selectivity, regeneration and effective removal rate at neutral pH levels. In this study, we discussed the reasons of the high arsenic adsorption from groundwater of our previously developed adsorbent, a cationic polymer gel, *N,N*-dimethylamino propylacrylamide, methyl chloride quaternary (DMAPAAQ), loaded with iron hydroxide. We used a transmission electron microscope (TEM) and thermogravimetric analyser (TGA) to detect the iron contents in the gel and ensure its maximum impregnation. We found that the gel contains 62.05% FeOOH components. In addition, we used the Mössbauer spectroscopy to examine the type of impregnated iron in the gel composite and found that it was γ-FeOOH. Finally, we used Fourier transform infrared spectroscopy (FTIR) to examine the surface functional groups present in the gel and the differences in those groups before and after iron impregnation. Similarly, we also investigated the differences of the surface functional groups in the gel, before and after the adsorption of both forms of arsenic. To summarize, this study described the characteristics of the gel composite, which is selective in adsorption and cost effective, however further applications should be investigated.

## Introduction

Arsenic (As) is a toxic element, and if consumed in drinking water, can cause various chronic diseases such as pneumonia^1^, diabetes, hypertension, cancer, skin lesions. In addition it severely damages the digestive, circulatory, neurological and respiratory system^2–6^. Almost 200 million people in 105 countries suffer from chronic diseases caused by long term exposure and consumption of contaminated water^7^. Long term exposure may also result in loss of life.

In the environment, there are many types of arsenic. Mainly, two types of inorganic oxyanions of arsenic are dominant in contaminated water. They are As(III) and As(V)^8^. In the environment, there are many sources of arsenic. Natural processes such as weathering reactions, anthropogenic and biological activities, and volcanic emissions mobilize arsenic in the environment^9^. Arsenic can therefore be found in the atmosphere, soils, rocks, natural waters and organisms^10,11^. Of all these sources, human beings are exposed to high levels of arsenic through the consumption of groundwater^12^. The dominant types of arsenic found in contaminated water are As(III) and As(V)^8^, two inorganic oxyanions of arsenic.

The World Health Organization (WHO) has set the maximum limit of arsenic contamination in drinking water at less than 0.01 mg/L^13,14^. However, this limit is exceeded in the groundwater in almost twenty-one countries including USA, China, Chile, Taiwan, Mexico, Argentina, Poland, Canada, Hungary, Japan and others. Among these 21 countries, Bangladesh and India have the highest levels of arsenic contamination in groundwater^15,16^.

Particularly in the case of Bangladesh, water is contaminated by natural arsenic found in the minerals that flows from the Himalayas into groundwater^17^. People depend on this groundwater for drinking and daily usage. According to WHO, the arsenic contamination is so high in Bangladesh that it “is the largest mass poisoning of a population in history”^18–20^. To improve this situation arsenic removal technology is urgently needed.

Previously, researchers and scientists have developed different arsenic removal methods^21^ including coagulation, precipitation^22^, ion-exchange^23–26^, adsorption, reverse osmosis, ultrafiltration, electrochemical treatments^24^, combinations of adsorption and membrane technology^27^ and others^28–31^. Many researchers agree that adsorption is one of the best techniques because of its user friendliness, cost efficiency and overall effectiveness^15,32–40^. Activated carbons are one of the most frequently used adsorbents. However, they only function at very narrow pH ranges and also the amount of adsorption of both As(III) and As(V) is low^32^ as well.

P(3-acrylamideopropyl) trimethyl ammonium chloride, which is a cationic cryogel was prepared and a 96% of removal rate was achieved^41^. Iron oxyhydroxide powders are also contemplated as effective adsorbents^42,43^. Recently, researchers utilized several types of polymer gels for instance cryogels, microgels, cationic hydrogels, etc., to adsorb arsenic. These gels exhibited competent adsorption properties. For example, the cationic cryogel, poly(3-acrylamidopropyl) trimethyl ammonium chloride [p(APTMACl)] achieved an arsenic removal rate of 96%^41^. Additionally, at pH 9, approximately 99.7% removal efficiency was achieved by this cationic hydrogel^44^. A microgel, tris(2-aminoethyl) amine (TAEA) and glyceroldiglycidyl ether (GDE), p(TAEA-co-GDE) achieved 98.72 mg/g of maximum arsenic adsorption capacity was achieved by the microgel at pH 4^45^. Although these gels have high adsorption capabilities, their selectivity in all studied environments was low and they failed to effectively remove arsenic from water at neutral pH levels^11^. A maximum adsorption capacity of 227 μg/g of was measured when Fe(III)-Sn(IV) mixed binary oxide-coated sand was used at a temperature of 313 K and a pH of 7^46^. Alternatively, Fe-Zr binary oxide-coated sand (IZBOCS) has also been used to remove arsenic and achieved a maximum adsorption capacity of 84.75 mg/g at 318 K and a pH of 7^47^. The core shell Fe@Fe_2_O_3_ nanobunches (NBZI) removed arsenic from acidic wastewater by adsorption and co-precipitation. But, since this technique produces sediments, additional separation processes are required^48^.

Other reported adsorbents exhibited low adsorption performances, lack of recyclability, low stability, high operational and maintenance costs, and the use of hazardous chemicals in the synthesis process^35^. Therefore, researchers need to develop a technology that can remove arsenic efficiently at neutral pH levels, provide selectivity, cost effectiveness and can be reused.

A composite of cationic polymer gel loaded with iron hydroxide was discussed to provide better selectivity, reusability, and high arsenic adsorption. The gel used composite of *N,N*-dimethylamino propylacrylamide, methyl chloride quaternary (DMAPAAQ) and Iron(III) Hydroxide (FeOOH) particles. This gel can effectively adsorb arsenic at neutral pH levels, something most of the previous researches could not attain. The gel follows pseudo 2^nd^ order reaction kinetics, which suggests that the adsorption type was chemisorption. The maximum arsenic adsorption capacity of the gel was 123.4 mg/g, at neutral pH levels. In our previous research, we reported that the adsorption amount of As(V) by DMAPAAQ + FeOOH gel composite was higher than the other researches at neutral pH levels^11^. This gel functions when it is put into water and its surface area increases to adsorb arsenic. Inside the gel, a cationic charge (quaternary amino group) adsorbs arsenic and the iron hydroxide found in this gel improves selective adsorption. The arsenic adsorption mechanism showed that 64.4% arsenic was adsorbed by FeOOH components, whereas 35.5% was adsorbed by the protonated amino group of DMAPAAQ + FeOOH gel. The type of arsenic adsorption in the structure of the gel composite was ‘chemisorption’ as it followed pseudo 2^nd^ order reaction kinetics. The gel selectively adsorbed arsenic with SO_4_^2−^ solution. Notably, 87.6% regeneration efficiency over the course of eight consecutive days of experimentation was achieved, when NaCl was used for desorption, instead of harmful NaOH. The use of NaCl for desorption of As(V) from an adsorbent was an advantageous finding^11,49^. In the present study, we evaluated the characteristics that contribute to the high adsorption performances of the gel composite and discussed the details in the later sections.

## Results and Discussion

### The presence of FeOOH particles in the gel composites

**Figure 1:**
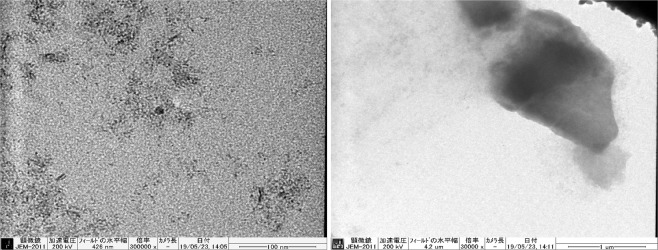
TEM images of DMAPAAQ + FeOOH gel composite (Scale bar length: left 100 nm, right 1 µm).

After preparing the gel composite, we analysed whether the gel contained iron particles. The diameter of the FeOOH particles of DMAPAAQ + FeOOH and DMAA + FeOOH composites were evaluated using a Transmission Electron Microscope (TEM-2010, JEOL Ltd., Japan). We found the presence of FeOOH particles in DMAPAAQ + FeOOH and DMAA + FeOOH gel composites after examination. TEM images of DMAPAAQ + FeOOH are shown in Fig. 1. In Fig. 1, the nano sized particles were clearly visible. In the right image of Fig. 1, conglomerated FeOOH particles in the structure of DMAPAAQ gel was visible. When the image was further magnified, the presence of the FeOOH particles of about 6–13 nm could be confirmed (from the left image of Fig. 1). Similarly, in the structure of the non-cationic polymer gel composite, DMAA + FeOOH, similar components of FeOOH were confirmed (Fig. S1). These results validate the preparation method described in Safi *et al*.^11,49^ and confirm that FeOOH components are successfully impregnated. The impregnation of the polymer gel structure with FeOOH is important because it increases the adsorption efficiency of the adsorbents for both As(III) and As(V)^50^. Since, both the FeOOH particles and the polymer gel structure can adsorb arsenic, we show that the presence of the FeOOH particles helps to increase the capability of adsorption of arsenic.

Therefore, we consider that arsenic is likely to be incorporated in the FeOOH particles and also in the polymer structure of DMAPAAQ gel. This shows that high adsorption ability can be achieved by combining two adsorbents in one gel composite.

**Figure 2:**
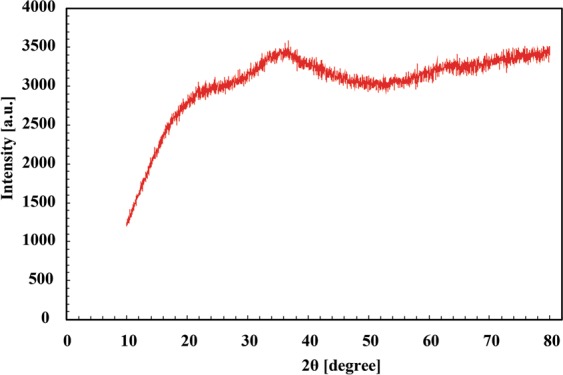
XRD analysis of DMAPAAQ + FeOOH gel composite.

We also analyzed the polymer structure of the DMAPAAQ + FeOOH gel composite by conducting an X-ray diffraction system. The result of the XRD analysis is shown in Fig. 2. The figure suggest that the diffractograms of DMAPAAQ + FeOOH samples show that the crystalline phase of DMAPAAQ + FeOOH was not developed. Hence, it suggests that the polymer structure of the gel is amorphous and it shows no crystallinity in the structure^51^. The TEM images also showed no sign of crystallinity of the particles or polymer in the gel structure. The polymer structure of DMAPAAQ + FeOOH is highly crosslinked between the DMAPAAQ gel and FeOOH particles.

### Content of FeOOH particles in the gel composite

**Figure 3:**
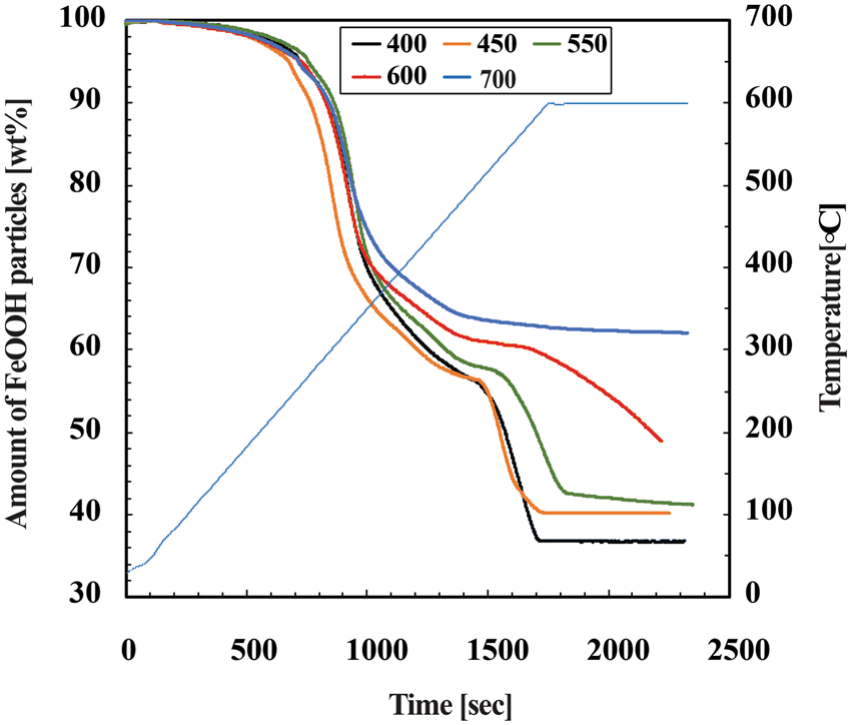
Thermogravimetric analysis curves to analyse the Content of FeOOH particles in DMAPAAQ + FeOOH gel composite.

The volume of FeOOH in the gel composite may affect the ability of the gel composite to adsorb arsenic. Moreover, the higher the volume of FeOOH in the gel composite, the higher the selectivity of arsenic adsorption. In addition, maximum adsorption of arsenic can be obtained. As described in the ‘Methods’ section, to achieve the highest content of FeOOH inside of the gel composite, FeCl_3_ and NaOH concentrations were varied to a ratio of 1:3 during the preparation of the gel. One of the novelties of our unique preparation method is that FeCl_3_ was added in the initiator solution and NaOH was added with the monomer solution so that when the two solutions are mixed, FeOOH is produced by the reaction between FeCl_3_ and NaOH, at the preparation stage. Hence, this method will ensure that the gel contains the highest volume of FeOOH. DMAPAAQ + FeOOH gel was prepared with different concentrations of FeCl_3_ (400, 450, 550, 600 and 700 mM/L)_._ Similarly, a non-cationic gel composite, DMAA + FeOOH gel was developed with different concentrations of FeCl_3_ (450, 550, 650 and 700 mM/L), for comparison purpose_._ The content of the FeOOH particles in the gel composites of DMAPAAQ + FeOOH and DMAA + FeOOH at different FeCl_3_ concentrations was measured by Thermogravimetric Analyzer (TGA-50, Shimadzu Co., Japan). We suspect that most of the Fe ions have formed iron hydroxide inside of the gel. Figure 3 shows that when the FeCl_3_ concentration was 700 mM / L, the content of the particles was highest (62.05 wt%). The result was confirmed by the TGA curves shown in Fig. 3, where the TGA curves were shown for the different concentrations of FeCl_3_ (400, 450, 550, 600 and 700 mM/L), in the structure of DMAPAAQ + FeOOH gel.

On the other hand, when the concentration of FeCl_3_ was set to more than 700 mM/L, formation of the DMAPAAQ + FeOOH gel composite became difficult. The reason for this difficulty is that when the concentration of NaOH was high, DMAPAAQ was hydrolysed and the formation of radicals by the initiator, ammonium peroxodisulfate (APS) was inhibited. As the maximum content of FeOOH was found at FeCl_3_ concentration of 700 mM/L, we chose this concentration to prepare the gel composites and conduct later experiments. In addition, Fig. S2 shows the relationship between the composition of charged iron ions and the content of FeOOH for DMAA + FeOOH gel composite. The figure suggests that the DMAA + FeOOH gel composite was able to obtain the similar content of FeOOH characteristic as DMAPAAQ + FeOOH gel composite.

### Identification of FeOOH particles

Arsenic adsorption property varies depending on different types of FeOOH. Hence, we performed Mössbauer spectroscopy to identify the type of FeOOH within the gel composite.

**Figure 4:**
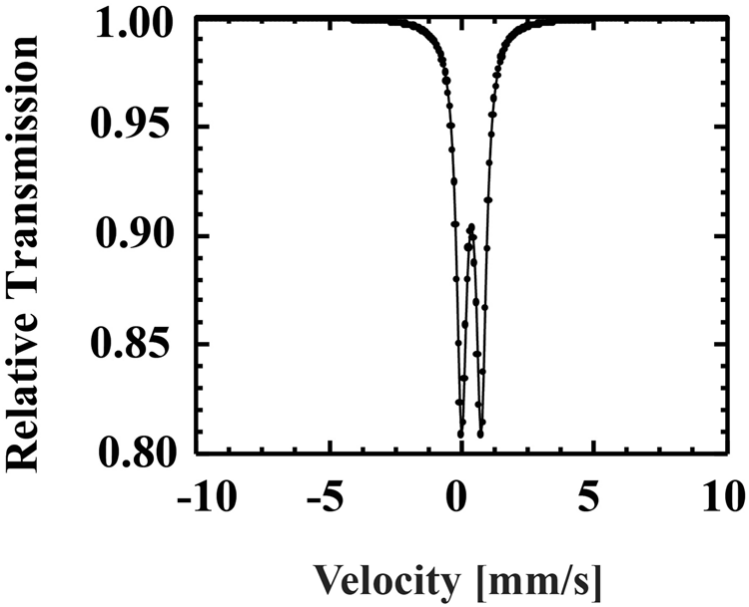
Mössbauer spectroscopy of DMAPAAQ + FeOOH gel composite.

So far, three different types of FeOOH particles such as α-FeOOH, β-FeOOH, γ-FeOOH have been observed^52^. As shown in Table. S1 and also in the previous section titled ‘Content of FeOOH in the gel composite’, our gel composite was prepared with 700 mM/L of FeCl_3_ and the highest content of FeOOH in our gel composite from TG analysis is 62.05 wt%. We experimented the type of FeOOH inside the structure of DMAPAAQ + FeOOH gel composite. The results of the Mössbauer spectroscopy of DMAPAAQ + FeOOH gel composite is shown in Fig. 4. In the figure, a quadrupole doublet was observed. After comparing our result with previous studies (e.g.^53,54^), we found that there is similarity between the peak of γ-FeOOH with the peak in the Fig. 3. Therefore, within the gel composite, FeOOH particles are γ-FeOOH.

This finding has a significant importance to rationale the high arsenic adsorption efficiency of the gel composite, DMAPAAQ + FeOOH. Repo *et al*.’s (2012) study suggests that γ-FeOOH can adsorb both As(III) and As(V) in high quantity^55^. Therefore, the presence of γ-FeOOH is one of the reasons for the high capability of arsenic adsorption efficiency by DMAPAAQ + FeOOH gel composite as shown in our previous study^11,49^.

### Surface functional group characterization using FTIR spectroscopy

#### FTIR spectra of DMAPAAQ gel, DMAPAAQ + FeOOH and DMAA + FeOOH gel composites

**Figure 5:**
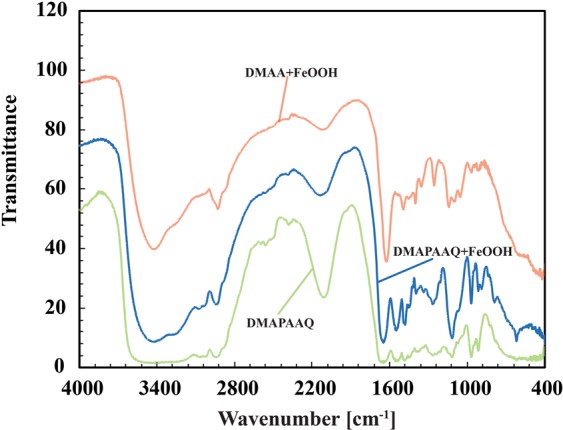
FTIR spectroscopy of DMAPAAQ gel (light green line), DMAPAAQ + FeOOH (blue line) and DMAA + FeOOH gel composite (pink line).

We compared the spectral peaks of the DMAPAAQ gel, DMAPAAQ + FeOOH and DMAA + FeOOH gel composites, to determine the difference in surface functional group characterization, before (light green line in Fig. 5) and after (blue line in Fig. 5) the impregnation of DMAPAAQ gel structure with FeOOH. The FTIR spectrum of non-ionic gel composite of DMAA + FeOOH (pink line in Fig. 5) was examined for the purpose of comparing ionic and non-ionic gel composite characteristics. As Fig. 5 and Table S2 shows, the FTIR spectrum of DMAPAAQ + FeOOH gel (blue line), the spectral bands at 3417, 3282, 3064, 2596, 2561, 2112, 1643, 1485, 1382, 1336, 1269 and 623 cm^−1^ were shifted to 3414, 3277, 3074, 2945, 2598, 2567, 2144, 1649, 1481, 1396, 1338, 1267 and 619 cm^−1^,respectively, after the FeOOH impregnation to DMAPAAQ gel. We ascribed this to the vibration of the Aliphatic primary Amine N-H stretching, Alcohol O-H stretching, Carboxylic acid O-H stretching, Alkyne C≡C stretching, Alkene C=C stretching, Alkane C-H bending, Aldehyde C-H bending, and Aromatic ester C-O stretching groups. As shown in Table S2, these shifts contribute to the change in groups. In addition, the spectral peak at 914 cm^−1^ (Alkene C=C bending) disappeared and new peaks appeared at spectral band 889 (1,2,4-trisubstituted C-H bending) and 788 cm^−1^ (1,2,3-trisubstituted C-H bending). Spectral bands at 2522 (Carboxylic acid O-H stretching), 1116 (secondary Alcohol C-O stretching) and 968 cm^−1^ (Alkene C=C bending) remained the same for both DMAPAAQ gel and DMAPAAQ + FeOOH gel composite.

In the case of the non-cationic gel composite, DMAA + FeOOH (pink line), the spectral peaks appeared at wavelengths 3433 (Aliphatic primary Amine N-H stretching), 2929 (Amine salt N-H stretching), 2123 (Alkyne C≡C stretching), 1625 (Alkene C=C stretching), 1494 and 1454 (Alkane C-H bending), 1400 (Aldehyde C-H bending), 1355 (Alcohol O-H bending), 1255 (Aromatic ester C-O stretching), 1141 (tertiary Alcohol C-O stretching), 1099 (secondary Alcohol C-O stretching), 968 and 906 cm^−1^ (Alkene C=C bending).

The above analysis suggests that there are differences in surface functional characteristics after loading FeOOH in DMAPAAQ gel structure. Some of the groups found in DMAPAAQ + FeOOH gel composite are: strong Alcoholic and Carboxylic acid O-H stretching group, primary Amine N-H stretching group, Alkane C-H bending, Alkene C=C stretching, Aldehyde C-H bending, Aromatic ester C-O stretching, and strong Alkene C=C bending group. After the FeOOH impregnation in DMAPAAQ gel, 1,2,4- trisubstituted strong C-H bending group and Alkyne C≡C stretching group were generated in the structure.

#### FTIR spectra of As(III) and As(V) loaded DMAPAAQ + FeOOH gel composites

**Figure 6:**
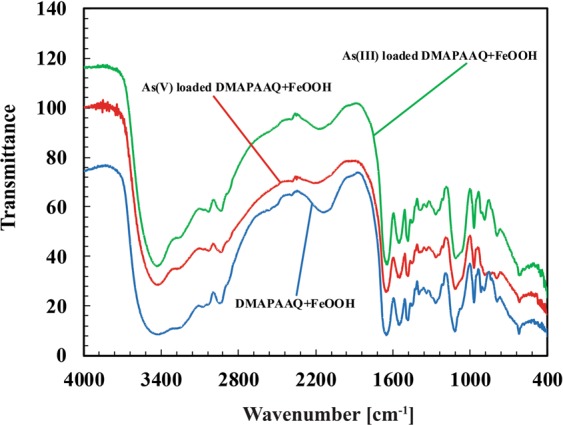
FTIR spectroscopy of DMAPAAQ + FeOOH gel composite (blue line), As (III) loaded (green line) and As (V) loaded DMAPAAQ + FeOOH gel composite (red line).

The FTIR spectra of As(III) and As(V) loaded DMAPAAQ + FeOOH gel composite revealed a shift in the position of some spectral peaks. Figure 6 and Table S2 shows the FTIR spectral bands of DMAPAAQ + FeOOH (blue line), As(III) loaded (green line) and As(V) loaded DMAPAAQ + FeOOH (red line). In the As(III) loaded DMAPAAQ + FeOOH gel composite (green line in Fig. 6), the spectral bands at 3414, 3074, 2945, 2144, 1396, 1267, 1116, 889 and 619 cm^−1^ were shifted to 3433, 3066, 2935, 2160, 1382, 1265, 1110, 885 and 615 cm^−1^, respectively, after the As(III) impregnation of DMAPAAQ + FeOOH gel composite. We ascribed this to the vibration of the Aliphatic primary Amine N-H stretching, Alcohol O-H stretching, Amine salt N-H stretching, Alkyne C≡C stretching, Aldehyde C-H bending, Aromatic ester C-O stretching, Secondary Alcohol C-O stretching, and 1,2,4-trisubstituted C-H bending groups.

Similarly, in the As(V) loaded DMAPAAQ + FeOOH gel composite (red line in Fig. 6), the spectral bands at 3414, 2945, 2144, 1649, 1396, 1116, 889, 788 and 619 cm^−1^ were shifted to 3421, 2933, 2212, 1643, 1388, 1114, 885, 790 and 615 cm^−1^ respectively after the adsorption of DMAPAAQ + FeOOH gel with As(V). We ascribed this to the vibration of the Aliphatic primary Amine N-H, Amine salt N-H stretching, Alkyne C≡C stretching, Alkene C=C stretching, Aldehyde C-H bending, secondary Alcohol C-O stretching, 1,2,4-trisubstituted C-H bending, and 1,2,3-trisubstituted C-H bending groups. New spectral peaks appeared at 2326 and 2347 cm^−1^ (Carbon di oxide O=C=O stretching).

In both the As(III) and As(V) loaded DMAPAAQ + FeOOH gel composites, the spectral peaks at 2598, 2567 and 2522 cm^−1^ (Carboxylic acid O-H stretching) disappeared. The spectral bands at 3277 (Alcohol O-H stretching), 1481 (Alkane C-H bending), 1338 (Alcohol O-H bending), 968 cm^−1^ (Alkene C=C bending) were the same for the DMAPAAQ + FeOOH gel composites, As(III) and As(V) loaded DMAPAAQ + FeOOH gel composites.

The analysis suggests that ion exchange and surface complexation reactions occurred in the gel composite. The shifts in the spectral bands denote that As(III) and As(V) changed the surface functional characteristics of DMAPAAQ + FeOOH gel composite, by being highly adsorbed in the structure of the gel composite. Some of the groups that were found in As(III) and As(V) loaded DMAPAAQ+FeOOH gel composites are: strong alcoholic and carboxylic acid O-H stretching group, primary Amine N-H stretching group, Alkyne C≡C stretching, Alkene C=C stretching, Alkane C-H bending, Aldehyde C-H bending, Aromatic ester C-O stretching, secondary alcohol C-O stretching, strong Alkene C=C bending. After the loading of As(V) in DMAPAAQ gel, Carbon dioxide (O=C=O stretching) was generated in the gel structure.

Referring to the “Arsenic adsorption isotherm” section from our previous research paper, the maximum capability of adsorption of arsenic by DMAPAAQ + FeOOH was higher when compared with the other currently studied methods at neutral pH levels. The higher adsorption capability has strong correlation with the FTIR analysis because the N-H stretching groups shift due to the adsorption of both As(III) and As(V) to DMAPAAQ + FeOOH. Furthermore, a change (disappearance) was observed in O-H stretching groups after the impregnation of both As(III) and As(V) to DMAPAAQ + FeOOH. The arsenic adsorption mechanisms discussed in our previous paper also supports the above hypothesis. Hence, we believe that, the FTIR analysis can describe why DMAPAAQ + FeOOH has a higher Q_max_ value (maximum adsorption capacity) than the other adsorbents recently studied^11^.

#### X-Ray Photoelectron Spectroscopy (XPS) analysis of Arsenic adsorption on DMAPAAQ + FeOOH gel

**Figure 7:**
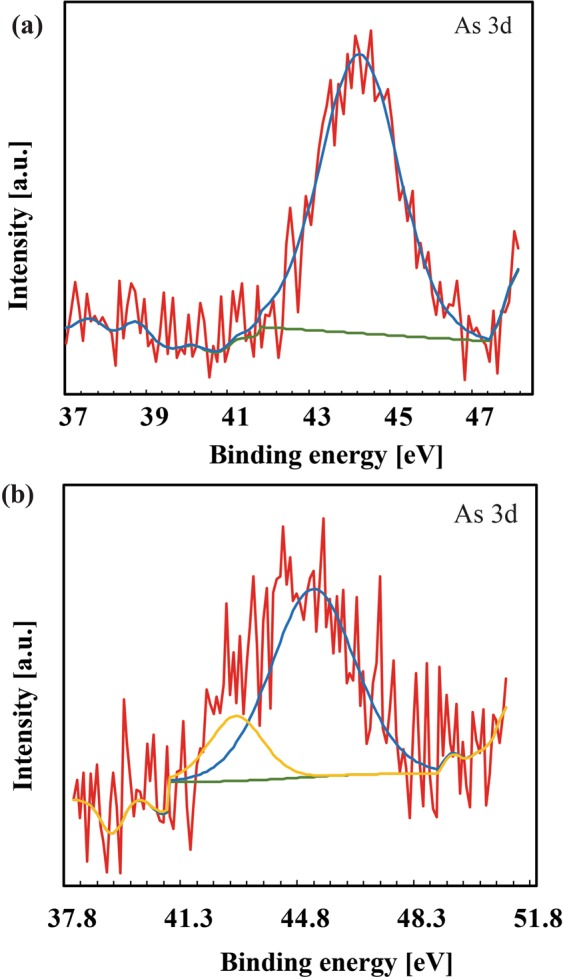
XPS at As3d peak of DMAPAAQ + FeOOH gel (**a**) reacted with As(III), (**b**) reacted with As(V).

The surface of DMAPAAQ + FeOOH gel composite was examined using XPS to verify the presence of As(III) and As(V). Two different samples were analyzed with XPS. One sample consisted of DMAPAAQ + FeOOH gel adsorbed with 50 mg/L of As(III) solution (Fig. 7(a)). And the other sample was DMAPAAQ + FeOOH gel adsorbed with 50 mg/L of As(V) solution (Fig. 7(b)). The results of XPS analysis of both the samples are shown in Fig. 7(a,b). We found that the samples of DMAPAAQ + FeOOH gel reacted with arsenic and generated As3d core level peaks.

In the both figures of Fig. 7, the XPS of the As3d peaks indicate the presence of arsenic on the surface of DMAPAAQ + FeOOH gel composite. The XPS spectrum of As 3d confirmed the coexistence of As (III) and As (V) in the structure of the polymer gel composite. The As3d binding energies were 44.23 and 45.28 eV for samples reacted with As(III) and As(V), respectively.

As(III) binding energies are about 1 eV lower than that of As(V). Also, the shape of the peak notifies the presence of a single species contributor. When arsenic was adsorbed to cupric oxide nanoparticles, similar results were obtained. Martinson and Reddy (2009) reported that in such cases, adsorption of As(III) involves a process of oxidation prior to adsorption^56^. In addition, similar findings were also reported when Fe-Mn binary oxide adsorbent was used to adsorb arsenic^57^ and when arsenic was adsorbed by Zeolite supported nanoscale zero-valent iron^58^.

Therefore, the oxidation of As (III) to As (V) occurred in the adsorption process of arsenic by DMAPAAQ + FeOOH gel composite.

## Conclusion

In the DMAPAAQ + FeOOH gel composite, the presence of iron particles was confirmed from the TEM images. The results from the TG analysis showed that DMAPAAQ + FeOOH gel composite contained 62.05% FeOOH particles in the gel composite. The high efficiency compared to the other adsorbents in our previous research paper may be because of the high content of FeOOH particles in the gel composite. The XRD analysis suggests that the polymer structure of the gel is amorphous. Mössbauer spectroscopy of DMAPAAQ + FeOOH gel composite suggests that the FeOOH particles present in the DMAPAAQ + FeOOH gel composite are γ-FeOOH, which aid in the adsorption of both As(III) and As(V). Therefore, γ-FeOOH contributed 64.4% of the adsorption of As(V) by DMAPAAQ + FeOOH gel composite. We calculated the adsorption amount of As(V) by the γ-FeOOH particles in the DMAPAAQ + FeOOH gel structure was 79.5 mg/g, which is almost 140% higher than that of the iron hydroxide powder^11,59^. Hence, it can be concluded that, γ-FeOOH particles enhanced the arsenic adsorption in the DMAPAAQ + FeOOH gel structure.

The FTIR analysis suggests that there were shifts in the surface functional groups due to the impregnation of FeOOH in the gel composite. In addition, shifts are apparent because of impregnation of As(III) and As(V) into DMAPAAQ + FeOOH gel composite. XPS analysis suggests that As(III) were oxidized to As(V) prior to being adsorbed by the DMAPAAQ + FeOOH gel composite.

There is further scope of research regarding the adsorption applications of As(III) to the gel composite. This study contributes to the field by examining and discussing the characteristics of an effective adsorbent of arsenic and the positive effects on contaminated water. All the characteristics in this study show that our gel composite, DMAPAAQ + FeOOH can solve the issue of arsenic contamination of groundwater not only by its high absorbance of arsenic, but also through its effective selectivity and regeneration.

## Methods

### Materials

The monomer, *N,N*′-dimethylamino propylacrylamide, methyl chloride quaternary (DMAPAAQ) (75% in H_2_O) was supplied by KJ Chemicals Corporation, Japan. The crosslinker, *N,N*′-Methylene bisacrylamide (MBAA) and the arsenic (III) sources, arsenic(III) oxide and sodium (meta) arsenite were purchased from Sigma-Aldrich, USA. The accelerator sodium sulfite (Na_2_SO_3_), the arsenic(V) source, disodium hydrogenarsenate heptahydrate (Na_2_HAsO_4_.7H_2_O) and ferric chloride(FeCl_3_) was purchased from Nacalai Tesque, Inc., Japan. Sodium hydroxide (NaOH) was purchased from Kishida Chemicals Corporation, Japan. *N,N*′- dimethyl acrylamide (DMAA) and the initiator, ammonium peroxodisulfate (APS) was purchased from Kanto Chemical Co. Inc., Japan.

### Preparation of the gel composite

The gel was prepared using the strategy meticulously described in Safi *et al*.^11,49^. We used *N,N*-dimethylamino propylacrylamide, methyl chloride quaternary (DMAPAAQ) as a monomer, *N,N*′-Methylene bisacrylamide (MBAA) as a crosslinker, sodium sulfite as an accelerator and ammonium peroxodisulfate (APS) as an initiator. Firstly, the monomer, DMAPAAQ and the crosslinker, MBAA, and the accelerator, sodium Sulfite and NaOH were composed and named as “monomer solution”. On the other hand, the initiator, APS and FeCl_3_ were mixed and named as “initiator solution”. The amount of NaOH and FeCl_3_ were taken on 3:1 proportion. The monomer and initiator solutions were first synthesized by N_2_ for 10 minutes at 10 °C. The solutions were then mixed together, and the reaction went for 4 hours at the same temperature (10 °C). The detailed composition of the gel composite is shown in Table S1. After the gel is formed, the block was first collected and washed with deionized water. Following this, the gel block was cut to obtain uniform square shaped pieces of gel composite of 5 mm in length. Finally, they were washed again with deionized water for 24 hours and dried in the oven for 24 hours at 50 °C. The purpose of adding NaOH and FeCl_3_ was to initiate the following reaction as well as to ensure the maximum content of Fe(OH)_3_ in the structure of the gel composite.

$${{\rm{FeCl}}}_{{\rm{3}}}+3{\rm{NaOH}}\to {\rm{Fe}}{({\rm{OH}})}_{3}+3{\rm{NaCl}}.$$

To evaluate the difference in terms of the characteristics of the cationic and non-cationic gel composite, *N,N*′*-*dimethyl acrylamide (DMAA) and FeOOH was prepared in the same way as DMAPAAQ + FeOOH gel composite.

### Transmission Electron Microscope (TEM) Analysis

The TEM images of the gel composite of DMAPAAQ + FeOOH and DMAA + FeOOH were analysed by Transmission Electron Microscope (TEM-2010, JEOL Ltd., Japan). We grinded 0.2 mg of DMAPAAQ + FeOOH gel to create fine powder, which was then immersed in 20 mL Isopropyl alcohol solution for 30 minutes. Following this, we collected 5 mL solution as a sample.

### Thermogravimetric (TG) Analysis

The examination of content of FeOOH in the DMAPAAQ + FeOOH and DMAA + FeOOH gel composite was carried out using a thermogravimetric analyser (TGA-50, Thermogravimetric Analyzer, Shimadzu, Japan). We took a piece of dry gel and cut it into a small piece to weigh less than 20 mg, with a stainless steel cutter. The small piece of gel was then placed in the titanium sample holder and the experiment was conducted at a heating rate of 5 °C/min from 50 °C to 600 °C in atmosphere. After testing each sample, the titanium holder was cleaned thoroughly using a clean cotton bud. We held the titanium holder with forceps to avoid heat.

### Mössbauer spectroscopy

The experimentation of the type of FeOOH was conducted using Mössbauer spectrometer (Wissel MB-500, Germany), with the help of Natural Science Centre for Basic Research and Development, Hiroshima University.

### Fourier transform infrared spectroscopy (FTIR) analysis

To examine the changes in the surface functional groups of DMAPAAQ gel after the FeOOH impregnation, we used the Fourier transform infrared spectroscopy (FTIR) (IR-Prestige 21, Shimadzu, Japan). In addition, we evaluated the changes in the surface functional groups of DMAPAAQ + FeOOH gel composite after the impregnation of As(III) and As(V). The concentration of As (III) and As(V) solutions were 0.2 mM. The solutions were prepared using distilled water as solvent. To adsorb arsenic, 20 mg of dried DMAPAAQ + FeOOH gel was immersed in 0.2 mM As (III) and As(V) solutions respectively for 24 hours at 25 °C. FTIR spectra of the DMAPAAQ gel, DMAPAAQ + FeOOH gel composite, DMAA + FeOOH gel composite and the As (III) and As (V) loaded DMAPAAQ + FeOOH samples were recorded. The wavelength range was from 400 cm^−1^ to 4000 cm^−1^. The samples were milled with potassium bromide (KBr) to form a very fine powder and then compressed into a thin pellet for analysis.

### X-Ray diffraction (XRD) analysis

The XRD analysis was conducted using MiniFlex 600 by Rigaku Corporation, Japan. We grinded 0.2 mg of DMAPAAQ + FeOOH gel into fine powder and placed the powder into glass film designated for XRD experiment.

### X-Ray photoelectron spectroscopy (XPS) analysis

The XPS analysis was conducted using an ESCA 3400 Electron spectrometer by Kratos Analytical Ltd., UK. We grinded 0.2 mg of DMAPAAQ + FeOOH gel into fine powder and placed the powder into glass film designated for XRD experiment. About 20 mg of dried DMAPAAQ + FeOOH gel pieces were immersed in 50 mg/L Na_2_HAsO_4_.7H_2_O and sodium (meta) arsenite solutions respectively. The solutions were then placed in a shaker for almost 9 hours at 25 °C and 120 rpm. The gels were then collected and dried in the oven at 70 °C for 12 hours. After drying, the gels were grinded with a mortar into fine powder, which was used for the experiment.

## Supplementary information

Supporting information

## Data Availability

The datasets analyzed during the current study are available from the corresponding author on reasonable request.
