# Supplementary material for: The effect of γ-FeOOH on enhancing arsenic adsorption from groundwater with DMAPAAQ + FeOOH gel composite
Source: Sci Rep. 2019 Aug 15;9:11909. doi: 10.1038/s41598-019-48233-x (PMC6695404; doi:10.1038/s41598-019-48233-x)
Supplement: Supplementary file 1 — Supporting information [file 41598_2019_48233_MOESM1_ESM.docx]

**­Supporting information**

**­The effect of γ-FeOOH on enhancing arsenic adsorption from groundwater with DMAPAAQ + FeOOH gel composite**

**Syed Ragib Safi^1^, Kiyotaka Senmoto^1^, Takehiko Gotoh^1,*^, Takashi Iizawa^1^ and Satoshi Nakai^1^**

^1^Department of Chemical Engineering, Hiroshima University, 1-4-1 Kagamiyama, Higashi Hiroshima, Hiroshima, Japan 739-8527

*tgoto@hiroshima-u.ac.jp

**Table S1.** Composition of gel composite.

|  | **Chemical** | **Quantity (mol/m^3^)** | |
| --- | --- | --- | --- |
| **Monomer** | DMAPAAQ, DMAA | | 500 |
| **Crosslinker** | MBAA | | 50 |
| **Accelerator** | Sodium Sulfite | | 80 |
|  | Sodium Hydroxide (NaOH) | | 2100 |
| **Initiator** | Ammonium peroxodisulfate (APS) | | 30 |
|  | Ferric Chloride (FeCl_3_) | | 700 |

| 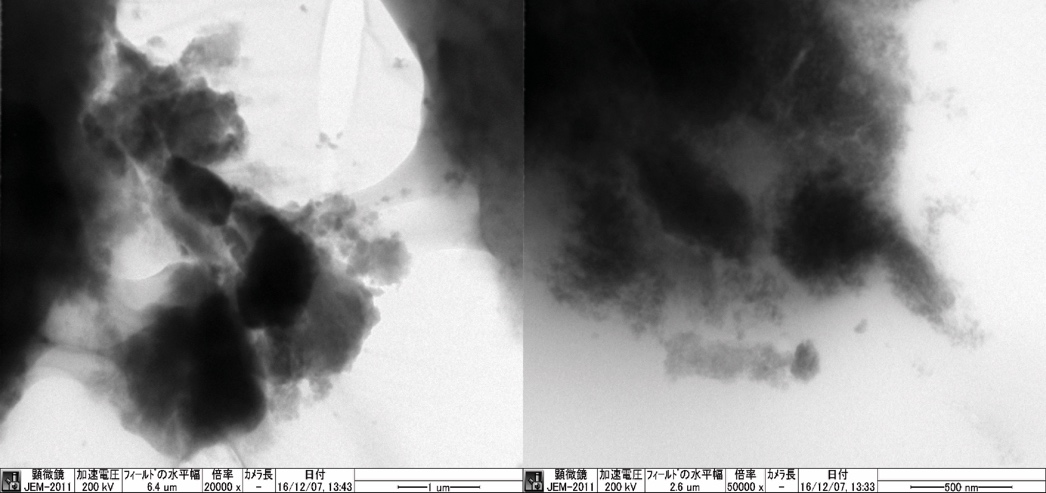  **Fig. S1.** TEM images of DMAA+FeOOH gel composite (Scale bar length：Left 1 µm, Right 500 nm). |
| --- |

|   **Table S2.** FTIR spectroscopy peak analysis.  **Fig. S2.** Thermogravimetric analysis to analyse the Content of FeOOH in DMAA+FeOOH gel composite. |
| --- |

| **Wavelength** | **DMAA+ FeOOH** | **DMAPAAQ** | **DMAPAAQ+ FeOOH** | **As(III) loaded DMAPAAQ+FeOOH** | **As(V) loaded DMAPAAQ+FeOOH** | **Group** | **Compound Class** | **Strength** |
| --- | --- | --- | --- | --- | --- | --- | --- | --- |
| 615 |  |  |  |  |  |  |  |  |
| 619 |  |  |  |  |  |  |  |  |
| 623 |  |  |  |  |  |  |  |  |
| 788 |  |  |  |  |  | C-H Bending | 1,2,3-trisubstituted | Strong |
| 790 |  |  |  |  |  |  |  |  |
| 885 |  |  |  |  |  | C-H Bending | 1,2,4-trisubstituted | Strong |
| 889 |  |  |  |  |  |  |  |  |
| 906 |  |  |  |  |  | C=C Bending | Alkene | Strong |
| 914 |  |  |  |  |  |  |  |  |
| 968 |  |  |  |  |  |  |  |  |
| 1099 |  |  |  |  |  | C-O Stretching | Secondary alcohol C-0 | Strong |
| 1110 |  |  |  |  |  |  |  |  |
| 1114 |  |  |  |  |  |  |  |  |
| 1116 |  |  |  |  |  |  |  |  |
| 1141 |  |  |  |  |  | C-O Stretching | Tertiary alcohol | Strong |
| 1255 |  |  |  |  |  | C-O Stretching | Aromatic ester | Strong |
| 1265 |  |  |  |  |  |  |  |  |
| 1267 |  |  |  |  |  |  |  |  |
| 1269 |  |  |  |  |  |  |  |  |
| 1336 |  |  |  |  |  | O-H Bending | Alcohol | Medium |
| 1338 |  |  |  |  |  |  |  |  |
| 1355 |  |  |  |  |  |  |  |  |
| 1382 |  |  |  |  |  | C-H Bending | Aldehyde | Medium |
| 1388 |  |  |  |  |  |  |  |  |
| 1396 |  |  |  |  |  |  |  |  |
| 1400 |  |  |  |  |  |  |  |  |
| 1454 |  |  |  |  |  | C-H Bending | Alkane | Medium |
| 1481 |  |  |  |  |  |  |  |  |
| 1485 |  |  |  |  |  |  |  |  |
| 1494 |  |  |  |  |  |  |  |  |
| 1625 |  |  |  |  |  | C=C Stretching | Alkene | Medium |
| 1643 |  |  |  |  |  |  |  |  |
| 1649 |  |  |  |  |  |  |  |  |
| 2112 |  |  |  |  |  | C≡C Stretching | Alkyne | Weak |
| 2123 |  |  |  |  |  |  |  |  |
| 2144 |  |  |  |  |  |  |  |  |
| 2160 |  |  |  |  |  |  |  |  |
| 2212 |  |  |  |  |  |  |  |  |
| 2326 |  |  |  |  |  | O=C=O Stretching | Carbon di oxide | Strong |
| 2347 |  |  |  |  |  |  |  |  |
| 2522 |  |  |  |  |  | O-H Stretching | Carboxylic acid | Strong, broad |
| 2561 |  |  |  |  |  |  |  |  |
| 2567 |  |  |  |  |  |  |  |  |
| 2596 |  |  |  |  |  |  |  |  |
| 2598 |  |  |  |  |  |  |  |  |
| 2929 |  |  |  |  |  | N-H Stretching | Amine salt | Strong, broad |
| 2933 |  |  |  |  |  |  |  |  |
| 2935 |  |  |  |  |  |  |  |  |
| 2945 |  |  |  |  |  |  |  |  |
| 3064 |  |  |  |  |  | O-H Stretching | Alcohol | Weak, broad |
| 3066 |  |  |  |  |  |  |  |  |
| 3074 |  |  |  |  |  |  |  |  |
| 3277 |  |  |  |  |  |  | Alcohol | Strong, broad |
| 3282 |  |  |  |  |  |  |  |  |
| 3414 |  |  |  |  |  | N-H Stretching | Aliphatic primary amine | Medium |
| 3417 |  |  |  |  |  |  |  |  |
| 3421 |  |  |  |  |  |  |  |  |
| 3433 |  |  |  |  |  |  |  |  |
